# Chitosan as a biomaterial for the prevention and treatment of dental caries: antibacterial effect, biomimetic mineralization, and drug delivery

**DOI:** 10.3389/fbioe.2023.1234758

**Published:** 2023-09-29

**Authors:** Shanlin Qu, Xiaolin Ma, Shuo Yu, Rui Wang

**Affiliations:** ^1^ Hospital of Stomatology, Jilin University, Changchun, China; ^2^ Jilin Provincial Key Laboratory of Tooth Development and Bone Remodeling, Changchun, China

**Keywords:** chitosan, dental caries, antibacterial, biomimetic mineralization, drug delivery

## Abstract

Dental caries is a chronic, progressive disease caused by plaque, influenced by multiple factors and can damage the hard tissues of the teeth. In severe cases, it can also lead to the onset and development of other oral diseases, seriously affecting patients’ quality of life. The creation of effective biomaterials for the prevention and treatment of dental caries has become one of the relentless goals of many researchers, with a focus on inhibiting the production of cariogenic plaque and retaining beneficial bacteria, guiding and promoting the reconstruction of dental hard tissues, and delaying the progression of existing caries. Chitosan is a natural cationic polymer extracted from the shells of crustaceans and shellfish. Since its discovery, chitosan has shown to have various biological functions such as antibacterial, biomimetic mineralization, drug delivery, *etc.*, making it one of the most promising biopolymers for new caries prevention and materials of prostheses. Therefore, this article provides an overview of the anti-caries applications of chitosan, which mainly covers the basic research on the application of chitosan in caries prevention and treatment since 2010, with a focus on categorizing and summarizing the following characteristics of chitosan as a caries prevention material, including its antibacterial effect, biomimetic mineralization effect and delivery ability of caries prevention drugs and vaccines. It also explores the limitations of current research on chitosan as a caries prevention biomaterial and the difficulties that need to be focused on and overcome in the future to provide theoretical reference for the clinical implementation of chitosan as a caries prevention biomaterial.

## 1 Introduction

Chitosan is the only positively charged polysaccharide in nature, a cationic polymer derived mainly from the chitin exoskeleton of marine crustaceans such as crabs and shrimps (Shoueir et al., 2021). Since its discovery by Rouget in 1859 (Raafat and Sahl, 2009), chitosan has attracted much attention and shown great promise in medicine and bioengineering. The components of chitosan are 2-acetamido-2-deoxy-β-d-glucopyranose and 2-amino-2-deoxy-β-d-glucopyranose. The lower the proportion of 2-acetamido-2-deoxy-β-d-glucopyranose, the higher the degree of deacetylation (DD) of chitosan, and the more free-amino groups it has, which is strongly related to the biological functions of chitosan, including its ability to carry drugs, bioadhesive characteristics, and antibacterial activity (Ali and Ahmed, 2018; Shoueir et al., 2021). Therefore, chitosan is also known as an amino polysaccharide. Additionally, the molecular weight (MW) of chitosan, which typically ranges from 10 to 1000 kDa (Yang et al., 2022), is another crucial factor that influences its biological activity (Raafat and Sahl, 2009). In addition to its unique biological activity, the biocompatibility, biodegradability and low cytotoxicity (Paradowska-Stolarz et al., 2021) of green-sourced chitosan are more conducive to expanding its application pathways in the fields of food, medicine and pharmaceuticals, in line with the concept of “green economy” (Mazur et al., 2022), and have significant and valuable research value.

**FIGURE 1:**
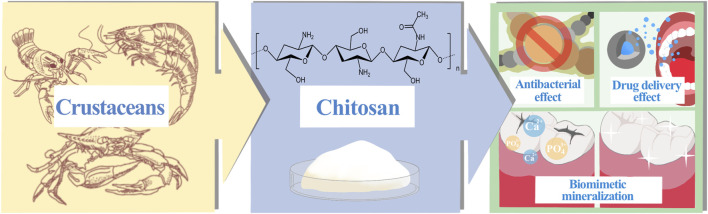
Crustacean-derived chitosan and its use in caries prevention.

Dental caries is a persistent, progressive disease that eats away at the hard tissues of the teeth. Severe caries can also lead to other oral diseases, such as pulpitis and periapical periodontitis (Galler et al., 2021; Huang et al., 2022), which can seriously damage the soft and hard structures of the oral cavity and are irreversible. Due to the uniqueness of the anatomical structure of the tooth’s hard tissue, its susceptibility to caries and the uncontrollability of each individual dietary habits, the global incidence of dental caries has remained high for many years, despite continuous improvements in socio-economic status and quality of life (Bernabe et al., 2020). Bacteria play a significant and critical role in the pathophysiology of dental caries. When they adhere to the tooth surface and interact constantly, they can form plaque, a complex ecological community with a largely stable microenvironment. At the same time, bacteria can ferment carbohydrates in the oral cavity to meet their own energy needs, and the acidic by-products of this process can lead to demineralization of tooth hard tissue (Sheiham and James, 2015). When there are high levels of sugars in the mouth, plaque continues to metabolize the sugars and produce acid, leaving the tooth surface in an irreversible state of demineralization for a prolonged period of time, gradually eroding the integrity of the tooth hard tissue. In essence, it is the disruption of the dynamic balance between dental minerals and oral bacteria that leads to the development of dental caries (Pitts et al., 2017; Krol et al., 2023). At present, the prevention and treatment of dental caries base on the following techniques: blocking or regulating the progression of dental caries by removing or managing the pathogens and restoring the balance of the oral microbiota; restoring the structure and function of teeth in response to the damage that caries has caused to their integrity (Cheng et al., 2022). Chitosan, as a relatively new natural polymer in dental applications (Yamakami et al., 2022), has been widely studied and confirmed for its anti-caries effects, mainly through the development of anti-caries products or clinical adjuvant treatment technologies, such as antibacterial, biomimetic mineralization and dentin binding, anti-caries drug delivery, with broad application prospects (Figure 1). As the first article we have learned about the anti-caries effect of chitosan, this article aims to provide a comprehensive overview of the anti-caries research of chitosan, summarize the research progress in the anti-caries mechanism of chitosan in recent years, and attempt to analyze the potential applications of chitosan in the future and the shortcomings of current research, providing theoretical reference for further researches and breakthroughs in chitosan anti-caries materials and clinical implementation.

## 2 Materials and methods

**FIGURE 2:**
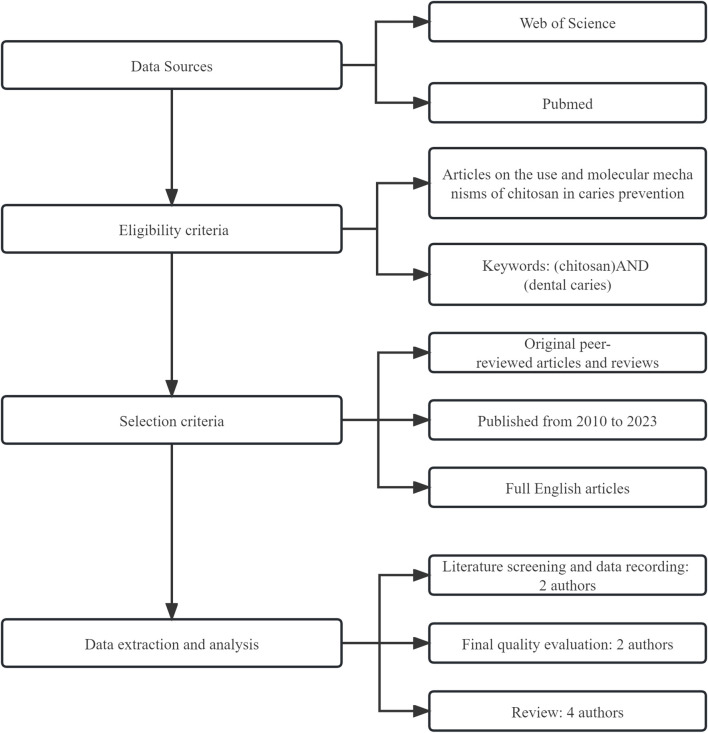
Article retrieval flowchart.

We searched the PubMed/MEDLINE and Web of Science databases for the most recent information on the use and mechanism of chitosan in caries prevention. The articles included are original peer-reviewed papers and reviews published since 2010, and papers without evident scientific backgrounds and prominent practical aspects were excluded. The search strategy for this article is shown in Figure 2.

## 3 The antibacterial effect of chitosan

### 3.1 Chitosan and its related materials inhibit oral cariogenic bacteria

At present, the antibacterial effect of chitosan has been preliminarily confirmed by a large number of experiments, and many studies have shown that chitosan has the ability to limit the growth of certain oral bacteria (Table 1), such as *Streptococcus mutans* (*S.mutans*) and *Porphyromonas gingivalis* (*P*.*gingivalis*). Because of this property, chitosan has been chosen by many researchers as a raw material for the development of oral health products. Evidence suggests that chitosan-containing products may have a significant therapeutic benefit in inhibiting plaque growth. For example, some researchers have developed a water-soluble chitosan mouthwash based on chitosan glucosamine. According to *in vitro* tests, the mouthwash has antibacterial properties equal to or better than other commercial mouthwashes, achieving 99.99% antibacterial activity by acting on all test strains for 20 s. Similar antibacterial properties have also been observed in *in vivo* experiments (Chen and Chung, 2012). Chewing chitosan chewing gum can also reduce the number of mutans streptococci in a person’s saliva, helping to prevent tooth decay (Khamverdi et al., 2021). Chitosan can also combine with other antibacterial materials or therapies to enhance the original antibacterial activity. One of the most effective ways to increase the antibacterial activity of chitosan is to modify it with metal ions (Ma et al., 2017). For example, compared to individual zinc oxide nanoparticles or chitosan, the antibacterial activity of chitosan-coated zinc oxide nanocomposites is dramatically increased (Javed et al., 2020). In addition, chitosan from a green source can reduce the cytotoxicity of metal ions (Maluin and Katas, 2022). Another promising antibacterial strategy is the combination of chitosan with photodynamic therapy (PDT). The ability of cationic chitosan to penetrate dense bacterial biofilms encapsulated in the extracellular matrix makes it a viable delivery vehicle for photosensitizers. Chitosan also has a physical inactivation effect on germs and can have synergistic antibacterial effects when combined with PDT (Guo et al., 2023). Based on sub-MIC concentrations of emodin-chitosan nanoparticles, antibacterial photodynamic treatment showed a significant reduction in *S. mutans* and ROS generation (Pourhajibagher et al., 2022). The proliferation of planktonic *S. mutans* and the development of biofilms can be significantly suppressed by antibacterial PDT mediated by chitosan and Photoditazine^®^ (de Souza et al., 2020).

**TABLE 1:** Antibacterial effects of chitosan with different molecular weights on some common oral bacteria (high molecular weight chitosan: HMW, low molecular weight chitosan: LMW, chitosan hydrolysate: CTSNs).

Chitosan	MW (kDa)	DD	Bacteria	Antibacterial effect	Ref.
HMW	624	>75%	*P.gingivalis*	MIC = 1 mg/mL	Costa et al. (2012)
				MBC = 5 mg/mL	
			*T.forsythensis*	MIC = 1 mg/mL	
				MBC = 5 mg/mL	
			*P. buccae*	MIC = 3 mg/mL	
				MBC = 7 mg/mL	
			*A.actinomycetemcomitans*	MIC = 5 mg/mL	
				MBC = 7 mg/mL	
			*P. intermedia*	MIC = 1 mg/mL	
				MBC = 5 mg/mL	
			*S. mutans*	MIC = 3 mg/mL	
				MBC = 5 mg/mL	
LMW	107	between 75% and 85%	*P.gingivalis*	MIC = 1 mg/mL	
				MBC = 3 mg/mL	
			*T.forsythensis*	MIC = 3 mg/mL	
				MBC = 7 mg/mL	
			*P. buccae*	MIC = 1 mg/mL	
				MBC = 3 mg/mL	
			*A.actinomycetemcomitans*	MIC = 3 mg/mL	
				MBC = 7 mg/mL	
			*P. intermedia*	MIC = 3 mg/mL	
				MBC = 7 mg/mL	
			*S. mutans*	MIC = 5 mg/mL	
				MBC = 7 mg/mL	
CTSNs	6.650	It is hydrolyzed from high molecular weight chitosan with a degree of deacetylation of 95% and the DD after hydrolysis is unknown	*S. mutans*	IC_50_ = 150 μg/mL	Lee and Park (2015)
			*MRSA*	IC_50_ = 230 μg/mL	
CTSNs-P	7.783		*S. mutans*	IC_50_ = 200 μg/mL	
			*MRSA*	IC_50_ = 220 μg/mL	
CTSNs-B	5.452		*S. mutans*	IC_50_ = 190 μg/mL	
			*MRSA*	IC_50_ = 240 μg/mL	
CTSNs-S	3.510		*S. mutans*	IC_50_ = 170 μg/mL	
			*MRSA*	IC_50_ = 210 μg/mL	

### 3.2 Chitosan’s antibacterial mechanism and current limitations

In relevant studies on the antibacterial mechanism of chitosan, the properties of MW and DD have been widely emphasized. The following principles, which are generally accepted by many experts, can outline the antibacterial hypothesis of chitosan based on the research already available: Firstly, several researchers have shown that low MW chitosan polymer chains are more flexible when binding to bacteria, which is an important factor in bacterial inactivation (Benhabiles et al., 2012); Secondly, low MW chitosan has the ability to pass through cell membranes, alter the shape of DNA and prevent the formation of biological macromolecules necessary for bacterial life (Sun et al., 2017; Khattak et al., 2019); In addition, chitosan with a higher level of deacetylation can carry more positive-charges through protonation, allowing it to adhere firmly to the surface of bacteria and have a better antibacterial effect (Sun et al., 2017); Furthermore, according to some researchers, chitosan may reduce bacterial pathogenicity by interfering with the production of virulence factors or molecules that facilitate intercellular communication by some pathogenic bacteria (Bano et al., 2017). However, according to some related researches, the above arguments may not fully capture the mechanism of action of chitosan on caries pathogens. Costa et al. (Costa et al., 2012) have determined the minimum inhibitory concentration (MIC) and minimum bactericidal concentration (MBC) of high MW chitosan (DD>75%, a MW of 624 kDa) and low MW chitosan (DD between 75% and 85%, a MW of 107 kDa) against various Gram-positive and Gram-negative bacteria. Interestingly, in this study, high MW chitosan and low MW chitosan showed different antibacterial effects on different bacteria, and the high MW chitosan even showed a lower MIC and MBC compared to the low MW chitosan for some bacteria, such as *S. mutans*, which is entirely inconsistent with previous conclusions. A similar conclusion in another experiment showed that high MW chitosan appeared to have more effective anti-caries pathogen properties than low MW chitosan (Abedian et al., 2020). Other academics noted that the disruption of cell walls or membranes is directly related to the antibacterial activity of chitosan. High MW chitosan has a greater inhibitory effect than low MW chitosan on mature *S. mutans* biofilm due to the interaction between its high positive-charge and the negative-charge of cell membranes, causing cell membrane disruption (Costa et al., 2013). However, the high positive-charge of this chitosan may be associated with its DD, and this study did not elucidate the specific reason. Although high MW chitosan has antibacterial properties, some researchers have hypothesized that its application is restricted by the low solubility caused by the high degree of polymerization. Several researchers have used chitosan enzymes to hydrolyze high MW chitosan (95% DD, 200 kDa) to produce various low MW chitosan hydrolysates with average MW ranging from 3.0 to 8.0 kDa and to study their biological functions. The results showed that many low MW chitosan can interfere with extracellular matrix development and bacterial growth by blocking the activity of dextransucrase (DSase), a critical point in the *S.mutans* polysaccharide production pathway. Although the exact process is unknown, it may be related to the electrostatic interaction between some *S.mutans* components and the hydrolyzed amino monosaccharides (Lee and Park, 2015). In conclusion, MW and DD affect antibacterial efficacy and applicability of chitosan. The antibacterial mechanism is not yet fully defined and the antibacterial effect may vary due to different charged molecular configurations on the surface of microbes (Bellich et al., 2016). Due to the specific nature of the oral microenvironment, it is impossible to generalize the results of previous comparable antibacterial tests. But it is undeniable that chitosan has significant antimicrobial activity.

Acid-producing and acid-tolerant bacteria in dental plaque can release acid on the surface of teeth and form a dense extracellular matrix to achieve high acid retention (Bowen et al., 2018). *S*.*mutans* is one of the typical representative strains of acid producing and acid resistant bacteria in plaque and has been extensively investigated by many scientists. *S.mutans* has been repeatedly cited over the years for its crucial role in promoting bacterial copolymerization and adhesion, plaque formation, the creation of an acidic microenvironment and the development of dental caries. It is one of the primary target bacteria chosen in several studies and the development of materials for caries prevention and antibacterial agents. Due to its greater capacity to absorb dietary carbohydrates, convert them into a number of acidic compounds, and maintain its homeostasis and proper growth in low pH conditions, *S. mutans* is of great concern (Lemos et al., 2019). However, the diversity of the internal microbiota in cariogenic plaque has been revealed in recent years thanks to the development of second-generation sequencing and metagenomic technology. The previous idea has been replaced by a new one, which suggests that multiple microorganisms acting together within the complex and diverse biofilm of dental plaque cause dental caries, rather than just the action of *S. mutans* alone (Simon-Soro and Mira, 2015; Sanz et al., 2017). Therefore, preventing and treating dental caries is not just a matter of monitoring and suppressing specific strains of bacteria. In recent years, the concept of caries prevention and treatment has shifted from widespread eradication of biofilms to targeted elimination of cariogenic plaque, retention of beneficial bacteria and maintenance of the ecological balance and stability of the oral microbiota. Therefore, at this stage, it would be pointless to blindly investigate the antibacterial function of chitosan. The antibacterial activity of chitosan may not be a “unique selling point” in terms of applicability to caries prevention, but rather a “bonus point”.

## 4 Biomimetic mineralization effect of chitosan

### 4.1 Chitosan’s guiding and mineralizing effect on enamel

Chitosan also has great potential in guiding and organizing the biomimetic mineralization of dental hard tissues (Table 2). Chitosan can inhibit demineralization of enamel and prevent the release of mineral ions, which is directly related to the barrier chitosan can create on the surface of teeth to prevent the infiltration of organic acids (Stamford Arnaud et al., 2010). In addition, demineralized enamel has a negative surface charge due to the significant loss of calcium ions, and chitosan has an affinity for demineralized enamel due to its positive-charge, which helps the adhesion and penetration into enamel of chitosan. Besides, for the long-term and stable occurrence of remineralization beneath the enamel surface, chitosan can prevent spontaneous precipitation on the enamel surface and encourage the ions necessary for mineralization to enter the deep lesion area of the enamel (Zhang et al., 2018a; Nimbeni et al., 2021). The carboxyl and hydroxyl groups of the chitosan chain can prevent the spontaneous precipitation of calcium phosphate by chelating calcium ions, which is essential for stabilizing ACP and promoting the production of its precursors (Chen et al., 2015; Hashmi et al., 2019). Based on the above properties, some scientists have developed a series of chitosan products to help remineralize tooth enamel and dental caries lesions. Simeonov et al. (2019) created a chitosan/calcium phosphate hybrid microgel using chitosan as a template for calcium phosphate deposition. On demineralized tooth specimens, the amorphous low crystalline calcium phosphate in the chitosan microgel can redissolve into ions and then deposite at the caries site to facilitate the nucleation and growth of calcium phosphate on the enamel model surface and promote the remineralization of caries lesions. Chitosan and modified chitosan (N-(2 (2,6-diaminohexanamide)-chitosan) have been fluorinated by using ion-interaction and have shown a strong inhibitory effect on the release of phosphate ions from the surface of hydroxyapatite. Fluorinated chitosan has a comparable effect to sodium fluoride but contains less fluoride ions, suggesting that it can reduce the dosage of fluoride required for treatment. It also has higher antibacterial activity and very low cytotoxicity (Rahayu et al., 2022).

**TABLE 2:** Research on chitosan in guiding and promoting biomimetic mineralization of dental hard tissues.

Chitosan related preparations	Main function	Ref
chitosan/calcium phosphate hybrid microgel	It can assist in the nucleation and growth of calcium phosphate on the surface of dental enamel models, promote remineralization of dental caries lesions and has good adhesion and antibacterial activity	Simeonov et al. (2019)
Fluorinated chitosan and its derivatives	Effectively inhibits the release of phosphate ions on the surface of hydroxyapatite, with an effect comparable to that of sodium fluoride	Rahayu et al. (2022)
Experimental-resin-based materials doped with carboxymethyl chitosan and calcium phosphate microfillers	Effectively induces biomimetic mineralization of collagen fibrils in dentin, effectively improving the durability of the resin-dentin bond	Huang et al. (2019)
Crosslinked chitosan-nanoparticles	Collagen fibers resistance to collagenase significantly increased	Kishen et al. (2016)

### 4.2 Chitosan’s guiding and mineralizing effect on dentin

In addition to promoting enamel remineralization, chitosan could also contribute to stabilization and direction of the biomimetic mineralization of dentin. Because the process of ACP conversion to apatite, which occurs spontaneously, is easily influenced by the pH of the solution (43), the early formation of apatite can easily prevent the penetration of minerals, which is not conducive to the occurrence of deep tissue remineralization and significantly reduces the mechanical stability of dentin. By chelating calcium ions, chitosan and its derivatives can prevent the early precipitation of ACP, which helps the deep collagen fibers to capture mineral ions and promote mineralization (Li et al., 2023). Type I collagen fibers are the main component of the dentin collagen matrix and have a unique physical structure that resembles a ‘gate’ allowing only molecules with a molecular weight of less than 6 kDa to enter while preventing molecules with a molecular weight of more than 40 kDa from entering (Price et al., 2009). This property has led some researchers to hypothesize that high MW carboxymethyl chitosan could blocked out of the collagen fibers and ACP could infiltrate into the collagen fibers through electrostatic interactions, which stimulates mineralization inside the collagen fibers and improves the mechanical properties of dentin, as well as inhibiting early crystallization and mineralization outside the fibers and stabilizing mineral precursors (Huang et al., 2019). In addition to increasing mineralization within collagen fibers, protecting collagen from degradation by collagenase is also the key to promoting biomimetic mineralization. Studies have shown that chitosan forms a cross-link with dentin type I collagen after carbodiimide cross-linking treatment to protect collagen and block the binding site of collagenase, preventing collagen destruction. Chitosan can also directly block collagenase in the absence of cross-linking agents (Kishen et al., 2016).

Some researchers have suggested that the addition of chitosan and its derivatives to resin-based materials or adhesives can increase the strength and stability of dentin bonds due to the intriguing biomimetic mineralization properties of these substances. The experimental resin for remineralization can suppress crystallization by releasing carboxymethyl chitosan while continuously releasing calcium and phosphorus ions to promote biomimetic mineralization of dentin (Huang et al., 2019). According to the research by Zhang et al. (2022) remineralization of the resin-dentin interface and improved permeability of hydrophobic monomers to the dentin matrix could be demonstrated following treatment with carboxymethyl chitosan solution, suggesting the potential for a novel type of indirect pulp capping agent. In recent years, external demineralization of dentin fibers has emerged as a new method for improving the stability of resin-dentin bonds. This method increases the durability of the dentin bond while preserving the integrity of the hydroxyapatite in the collagen fibers and preventing collagenase degradation. Pretreatment of the dentin bonding interface with chitosan (MW > 40 kDa) prior to bonding, according to Gu et al. (2019), is beneficial for the stability of the internal minerals of the dentin collagen and may also protect the collagen in the hybrid layer from protease activity. Chitosan has the potential to dramatically improve the integrity of the dentin bonding interface, as demonstrated by the significant reduction in water permeability of the resin-dentin hybrid layer and its antibacterial effect on three individual bacterial biofilms.

## 5 The drug delivery effect of chitosan

With a focus on prolonging the exposure of drugs to the oral environment, reducing drug loss and ensuring sustained drug release, chitosan has great application value in the delivery of various biological macromolecules or drugs. Chitosan can achieve drug delivery in a variety of forms, the most typical of which include composite materials such as films, microspheres, nanoparticles, nanofibers and nanocomposites (Ali and Ahmed, 2018). Besides, chitosan can also improve the clinical operability of some biosynthetic materials, such as injectable chitosan hydrogels (Zhu et al., 2019). As a biological macromolecule with antibacterial properties, chitosan can be used as a carrier for antibacterial drugs, while enhancing their ability to fight bacteria (Patil and Jobanputra, 2015). In addition, the positively charged amino groups carried by chitosan greatly assist in the attraction of the substance to the surface of mucous membranes or teeth, keeping the materials close to the tissue surface during the drug release process. Chitosan can deliver a range of anticaries substances, such as antibacterial metal ions, minerals, antibiotics, proteins, DNA, *etc.* However, some scientists have suggested that due to the influence of chitosan’s own properties (high hydrophilicity, high positive-charge, *etc.*), the encapsulation and slow release effects of chitosan hydrogels on cationic, hydrophobic or macromolecular substances are not ideal (Peers et al., 2020). Based on the above biological properties of chitosan, drug delivery systems related to chitosan mainly have the following administration routes, such as oral soft and hard tissue adhesion, mucosal delivery, enamel or dentin coating, *etc.* Some chitosan materials also achieve similar effects when incorporated into dental pulp sealants, solutions, scaffolds, restorations, adhesives or resins. Relevant research on chitosan for the delivery and prevention of caries-related agents is detailed in Table 3.

**TABLE 3:** Chitosan drug delivery system for anti-caries agents.

Chitosan drug delivery system	Delivery forms	Administration routes	Types of drugs	Anti-caries agents	Main function	Ref
Carboxymethyl chitosan nanogel	Nanogel	Enamel coating	Protein, minerals	Chimeric lysin ClyR and amorphous calcium phosphate	It can significantly reduce the colony count and biofilm activity of *Streptococcus* mutans and reduce enamel surface demineralization	Zhu et al. (2021)
An experimental adhesive resin with chitosan	Powder	Incorporating into resin	Antibiotic	Triclosan	It has long-term antibacterial activity and stability and can inhibit the formation and growth of *S.mutans* biofilm	Schauenberg Machado et al. (2019)
Chitosan-coated cellulose acetate phthalate-poloxamer enamel adhesive device	Nanocomposites	Enamel coating	Antibiotic	Minocycline	With superior mucosal and dental adhesion properties, it can achieve 8 h of continuous drug release and inhibit *S.mutans* biofilm	Singh et al. (2022)
Chitosan nanoparticles	Nanoparticles	Not mentioned	Metal ions, minerals	Zn–Nb bioglass-ceramic	Chitosan helps to form a strong bond between the composite material and tooth tissue, helping the composite material to maintain stability during ion release and remineralization processes to prevent secondary caries	Uskokovic et al. (2021)
Mucoadhesive electrospun chitosan-based nanofiber mats	Nanofiber	Adhesion to buccal mucosa	Plant active ingredients	Garcinia mangostana extract	It has good mucosal adhesion and releases antibacterial agents to inhibit *S.mutans* and *S.sanguis*	Samprasit et al. (2015)
Dual oral tissue adhesive chitosan nanofiber membranes	Nanofiber	Adhesion to oral hard tissue and soft tissue	Peptide	Antimicrobial Peptides	It has good tissue adhesion properties and can achieve pH-responsive drug delivery, inhibiting the activity of bacterial biofilms	Boda et al. (2020)
Chitosan hydrogel	Hydrogel	Enamel coating	Peptide	Amelogenin-derived peptide	It has pH-responsive properties and can release active ingredients in specific microenvironments, providing a dual caries-preventive effect of antibacterial and promoting remineralization of carious lesions	Ren et al. (2019); Ren et al. (2018)
Chitosan hydrogel	Hydrogel	Enamel coating	Protein	Amelogenin	The pH-responsive properties of chitosan can effectively release active ingredients, achieve antibacterial effects, promote remineralization of demineralized enamel and effectively prevent the occurrence of secondary caries	Ruan et al. (2013)
Chitosan/DNA complexes liposome nanoparticles	Conjugates	Vaccination of the nasal mucosa	DNA	Anti-caries DNA vaccine pGJA-P/VAX	It can effectively achieve nasal mucosal delivery of caries prevention vaccines, with high transfection rate and residence time, achieving long-term mucosal immunity	Chen et al. (2013)

### 5.1 Chitosan can serve as an effective vehicle for anticaries drugs

Chitosan carriers can achieve successful delivery of traditional antibacterial or mineralizing drugs. For instance, some researchers use carboxymethyl chitosan nanogel to carry the antibacterial chimeric lysin ClyR and ammonium chloride phosphate (ACP), which can achieve the dual anti-caries effect of antibacterial and mineralization promoting, and the effect is similar to that of chlorhexidine. The abundant carboxymethyl groups in carboxymethyl chitosan help to maintain the stability of ACP(52). Additionally, chitosan can significantly prolong the retention and efficacy of drugs. Schauenberg Machado et al. (2019) incorporated chitosan loaded with triclosan into the experimental resin, which further improved the antibacterial effect of triclosan, and this antibacterial effect can extended to 6 months with the help of chitosan. Besides, the positive-charge carried by chitosan gives it an affinity with the surface of oral soft and hard tissues, which helps to improve the retention and duration of the drug at the site of action. Research has shown that the chitosan-coated enamel bonding tool can deliver medication continuously for 24 h, slow the growth of *S.mutans* over time and adhere strongly to the tooth surface (Singh et al., 2022). Other researchers have used thiol-chitosan as mucosal adhesion polymers to create electrospun nanofibre mats (Samprasit et al., 2015), which generate disulfide bonds with mucus matrix glycoproteins by thiol groups to achieve strong adhesion between mats and mucous membranes (Frigaard et al., 2022). Antibacterial tests have shown that the mats has strong and sustained inhibition of *S*.*mutans* and *S. sanguinis in vitro*, as well as a reduction in the oral microbiota in the subjects’ bodies. Furthermore, other researchers have used chitosan as a matrix carrying bio-ceramic particles because the expandability of chitosan in physiological dentin solution and the electrostatic interaction between positively charged amino carried by chitosan and negatively charged silica and dentin collagen can form a close bond between the composite and tooth tissue, helping the composite to maintain stability during ion release and remineralization to prevent secondary caries (Uskokovic et al., 2021).

### 5.2 Chitosan has pH-responsive property

The available sugars in the plaque consumption environment on the tooth surface can lower the pH of the plaque microenvironment and, after reaching a certain level, enter the plateau stage, leading to demineralization of the tooth surface. When most of the available sugars in the environment consumed up, the pH rises and returns to the neutral state, and the enamel on the tooth surface enters the remineralization phase. The amount of sugar in the environment directly impact how long the platform period lasts. When the environment contains high levels of readily available sugars, dental caries develops because plaque continues to produce acid and the tooth surface remains in a hardly reversible state of demineralization for a prolonged period. In essence, the key to the cariogenic properties of plaque is the sustained low pH caused by high sugar levels (Zhang et al., 2018b). As a cationic polymer, chitosan contains many amino groups that can protonate in acidic conditions, causing chitosan to dissolve. However, in neutral and alkaline conditions, the solubility of chitosan is significantly reduced due to deprotonation (Jin et al., 2020). Therefore, chitosan has a pH-responsive property that also provides a very interesting entry point for its application of the prevention and treatment of dental caries. Based on this property, some scientists have developed a series of pH-responsive chitosan nanomaterials to target the inhibition and removal of cariogenic plaque. Chitosan-modified antibacterial nanomaterials can release antibacterial drugs by protonating the nanomaterials in an acidic environment (Boda et al., 2020), which has the effect of specific eliminating pathogenic plaque; in addition, the ability of chitosan nanomaterials with positive-charge due to protonation to bind with the negatively charged bacterial surfaces is enhanced in an acidic environment, making the antibacterial effect of nanomaterials in an acidic environment stronger than that in a neutral environment (Xu et al., 2022).

Apatite dissolves to release calcium and phosphorus ions when the pH of the tooth hard tissue falls below the threshold due to bacterial acid production. When the pH returns to neutral, the environmental mineral ions can redeposit on the tooth surface and promote mineralization and repair of tooth hard tissue. The pH of the dental microenvironment is critical to the repair of dental hard tissue. Even if a mineralizer is present in the environment, mineralization is difficult to achieve at low pH value. Therefore, selectively controlling the release of mineral ions using pH differences caused by tooth surface pathology and the physiological environment is beneficial for improving the retention rate of drugs in the oral cavity and achieving long-term drug stability. Researchers have developed several chitosan nanocomposites that are rich in remineralizing agents. Highly charged amino groups carried by chitosan can competitively capture hydrogen ions from acidic environments to form a positively charged protective layer that inhibits the process of pH decrease and prevents further penetration of organic acids and demineralization of tooth surfaces; in addition, chitosan interacts electrostatically to maintain stability and prevent degradation until the environment becomes neutral with biological macromolecules that regulate the synthesis of hydroxyapatite, such as amelogenin and its derived peptide. In neutral environment, chitosan has a weak positive-charge and low solubility and dissociates from the biological macromolecules it carries, promoting remineralization of enamel (Ruan et al., 2013; Ren et al., 2018; Ren et al., 2019).

### 5.3 Chitosan effectively promotes mucosal delivery and antigen transfection of vaccines to prevent caries

Chitosan can effectively transport anti-caries vaccines in addition to anti-caries drugs. Chitosan offers new perspectives and approaches for vaccine delivery and improving antigen transfection efficiency, although the debate about anti-caries vaccine research is ongoing. Positive-charge chitosan can protect DNA and promote its absorption by compressing DNA into nanocomposites. It can also deliver vaccines by attaching to the mucosal surface and altering epithelial permeability (Li et al., 2016). Some researchers have combined chitosan and anionic liposomes to develop a novel anti-caries DNA vaccine delivery nanoparticle with pH-responsive release and mucosal adhesion properties. Vaccine delivery can achieve through the nasal mucosa, which helps to prolong the vaccine retention time and enhance the nasal mucosal immune response (Chen et al., 2013). In addition, chitosan has a stimulatory effect on humoral and cell-mediated immune responses, so that it can be used not only as a delivery system but also as an immunostimulatory adjuvant for vaccine antigens. Bi et al. (2019) chose two adjuvant mixtures of chitosan-Pam_3_CSK_4_ (a TLR2 agonist) and chitosan-monophosphoryl lipid A (MPL, a TLR4 agonist) as immune response enhancers to increase and prolong the immune response induced by the recombinant *S. mutans* PAc protein. According to the experimental results, chitosan-Pam_3_CSK_4_ or chitosan-MPL can significantly increase the titer of PAc-specific antibodies in serum and saliva compared to Pac alone, which can reduce the severity of dental caries. However, some researchers have suggested that chitosan and its derivatives as vaccine adjuvants or delivery systems requires close attention to the potential cytotoxicity caused by their high surface positive-charges, efficacy and safety, and immune tolerance, which need a further assessment (Khademi et al., 2018).

## 6 Summary and prospect

In general, the anti-caries applications of chitosan can be summarized as follows: 1) Chitosan can effectively inhibit the development of biofilms and the growth of bacteria linked to caries, but the level of its antibacterial activity depends on the MW and DD of chitosan. The amino group carried by chitosan is the key to its antibacterial action, which can promote chitosan binding and even penetrate cell membranes, damaging bacterial genetic materials and biomacromolecules; The combination of chitosan and its derivatives with other antibacterial composite materials or methods can significantly improve the antibacterial effect on the original basis; 2) Chitosan can act as a reservoir for calcium and phosphorus ion deposition, which aids in the remineralization of enamel caries sites. In addition, high molecular weight chitosan and its derivatives with a molecular weight greater than 40 kDa can act as inhibitors of extracellular dentin collagen mineralization, promoting the orderly formation of mineralized crystals inside collagen and reducing collagenase’s activity to break it down; 3) Chitosan can serve as an efficient drug delivery vehicle for remineralizing or antibacterial agents, which helps to increase medication bioavailability, lower dosages, and preserve long-term treatment efficacy. In addition, the amino groups carried by chitosan are protonated in acidic environments, altering its solubility and conformation, giving it significant pH-responsive properties, further extending its application in the prevention and treatment of dental caries.

Chitosan has limited applications, mainly due to its poor solubility in water or other organic solvents. However, chitosan has a high potential for chemical modification due to a number of amino groups it carries (Abd El-Hack et al., 2020). Many scientists have modified chitosan in various ways to improve its physical and chemical properties. In addition to increasing solubility, modified chitosan has antimicrobial effects, improves drug absorption or has chelating properties. Furthermore, it is important to note that the majority of products used in the prevention and treatment of dental caries are liquid or semi-solid (Ahmadian et al., 2018) and rely on the coating of the hard tissues of the teeth to increase the convenience of application. Although chitosan has an affinity for the tooth surface, high concentrations of chitosan may limit the ability of the substance to spread over the tooth surface due to its high viscosity caused by the network of sugar chains created by chitosan’s hydrogen bonding and hydrophobic interactions (Calero et al., 2010; Pichaiaukrit et al., 2019). Studies have also shown that although the addition of chitosan derivatives to dental restorative materials or adhesives can help reduce bacterial accumulation at the repair interface and the likelihood of secondary caries, the resulting reduction in the mechanical properties of the composite material poses a significant challenge to the clinical implementation of chitosan (Stenhagen et al., 2019). Finally, it is critical to focus on the potential degradation of chitosan and its derivatives by salivary lysozyme and the potential effects of this lysozyme degradation (Wang et al., 2021). It is necessary to conduct scientifically designed animal and clinical studies to verify the role of chitosan *in vivo*. Although many researchers have developed anti-caries chitosan-based products, more supportive research is needed before these can be used in a clinical setting.
